# Chromatography and Mass Spectrometry: Evolving Techniques for Food Analysis

**DOI:** 10.3390/foods14152694

**Published:** 2025-07-30

**Authors:** Andreia Bento da Silva, Noélia Duarte

**Affiliations:** Research Institute for Medicines (iMed.ULisboa), Faculty of Pharmacy, Universidade de Lisboa, Avenida Prof. Gama Pinto, 1649-003 Lisboa, Portugal

## 1. Introduction

The assurance of food safety and quality is considered a worldwide concern due to its implications for public health. Amidst ongoing technological advances in food analysis, a growing number of compounds have been discovered as having a substantial impact on human health or in the organoleptic quality of food, even though they are frequently present at trace levels [1,2,3]. These recent discoveries have highlighted the need for the development of simple, sensitive, and accurate methodologies for food analysis, where gas chromatography (GC) and high-performance liquid chromatography (HPLC) combined with mass spectrometry (MS) play a distinct role [1,2,3,4]. These techniques have been subjected to extensive improvements, allowing for the detection of a growing number of aroma and bioactive compounds, as well as food contaminants, in a variety of complex food matrices [1,2,3].

Aiming at contributing to this emerging field, twenty-four manuscripts were submitted for consideration in this Special Issue (SI). After the rigorous *Foods* peer-review process, nine research articles were accepted for publication. These articles covered the main areas of application of food analysis, namely (a) the evaluation of food quality, as aroma and nutritional composition [contributions 1–6], (b) the detection of harmful residues which may have a detrimental impact on health [contributions 7–9], and (c) the development of predictive models for food quality and authenticity assessment [contribution 1,2,5].

In this editorial, a summary of each article will be presented, focusing on its application and on the methodologies employed, while emphasizing the main outcomes achieved. Lastly, the general conclusions and future approaches will be discussed.

## 2. Overview of Published Articles

### 2.1. Application of Chromatography and Mass Spectrometry in Food Analysis

A summary of each article published in this SI can be found in Table 1. Six manuscripts sought to evaluate food quality with respect to their aroma [contribution 1] and nutrient content, namely fatty acids [contributions 2,3], amino acids [contribution 1], monosaccharides [contribution 4], micronutrients [contribution 2], and phytochemicals [contributions 2,5,6].

Carabetta et al. [contribution 1] analyzed both aromatic compounds and amino acids in honey, aiming at developing a predictive model, as will later be discussed. Krusinski et al. [contribution 2] showed that beef fed with a biodiverse pasture diet (grass-finished beef) contained higher levels of bioactive compounds, including fatty acids, micronutrients, and phytochemicals, than those fed with a total mixed ration with and without 5% grapeseed extract supplementation. Maurício et al. [contribution 4] compared the fatty acid and lipid profiles of four edible *Chlorella vulgaris* strains and demonstrated that they can be selectively chosen as a source of added-value lipids to be used as ingredients in food and nutraceutical applications. Prasongdee et al. [contribution 6] argue that Thai basil extracts have potential as functional food ingredients with antioxidant and antithrombotic properties. Lastly, Zong et al. [contribution 4] studied the effect of different extraction procedures on monosaccharide composition of polysaccharides in their degradation under acidic hydrolysis and established optimal hydrolysis conditions.

Another pertinent aspect that warrants further attention is the presence of contaminants in foods, such as antimicrobials [contribution 7], pesticides [contribution 8], and potential carcinogens derived from food additives [contribution 9], which have been recognized as evolving and widespread problems [1,3,4]. Yévenes et al. [contribution 7] developed and validated an analytical method for the detection of antimicrobials in lettuce, which can be applied in exploring the transference of antimicrobial drugs from soil to the plant tissues. Nevistić and Tomas [contribution 8] investigated the matrix effects of more than 200 pesticide residues in four food matrices. Kim et al. [contribution 9] developed and validated a method for the detection of 1,4-dioxane, a suspected human carcinogen derived from food additives such as polyethylene glycol 600 (PEG 600), which can be employed in future food safety inspection assays.

Another evolving field in food analysis is the development of predictive models for food authentication and traceability [4,5,6]. Wu et al. [contribution 5] identified characteristic markers of Changping strawberries which might contribute to the development of strawberries with a pleasant fragrance and health benefits. Carabetta et al. [contribution 1] built a predictive model for correlating amino acid profiles to aromatic compounds in honey that can be used as an analytical tool for a rapid multicomponent analysis of food-quality indicators. Lastly, Krusinski et al. [contribution 2] showed that the finishing diet of cattle can be determined by analyzing the total omega-3 polyunsaturated fatty acids (PUFAs), micronutrients, and phytochemicals in beef, which are higher in grass-finished beef.

### 2.2. Emerging Tools in Food Analysis

In contemporary times, analytical techniques can be divided into two main types: targeted and nontargeted [1,5]. Targeted approaches allow the accurate and precise quantitation of the analytes of interest, while they do not consider the presence of other molecules for the analysis [1,5]. In contrast, nontargeted approaches are semiquantitative, but are able to detect all compounds that produce a signal with the detector employed [5].

High-performance liquid chromatography (HPLC) is widely employed in food analysis for the quantitation of natural food constituents, food additives, toxins, and chemical residues, among others [4]. In fact, HPLC techniques with more cost-friendly detectors are still widely employed in targeted food analysis for the quantitation of natural food constituents. For instance, Krusinski et al. [contribution 2] quantitated vitamin E in beef by HPLC-UV, whereas Prasongdee et al. [contribution 6] employed HPLC with diode array detection (DAD) for the analysis of chlorophyll and carotenoid contents of Thai basil extracts, as well as for the evaluation of their phenolic profiles. However, the integration of chromatographic separation with mass spectrometry (MS) has significantly improved the analytical performance of these methods, allowing the analysis of several compounds in complex food matrices with improved specificity, sensitivity, and selectivity [1,2,3,4,7]. Notably, HPLC coupled to tandem mass spectrometry (MS/MS) is considered the most established technique for food analysis [2]. Yévenes et al. [contribution 7] developed an HPLC-MS/MS method for the detection of antimicrobials in lettuce. Ultra-high performance liquid chromatography (UHPLC) has the advantage of significantly enhancing chromatographic resolution and sensitivity, reducing analysis time [4]. Carabetta et al. [contribution 1] developed a UHPLC-MS/MS method for the analysis of amino acids, and Wu et al. [contribution 5] analyzed the phenolic compounds of strawberries by UHPLC-DAD-MS/MS.

However, HPLC-MS/MS analysis faces limitations such as reduced analyte scope and labor-intensive method development [2]. High-resolution MS surpasses the low-resolution MS in a nontargeted approach by allowing full-scan acquisition with superior sensitivity [2]. Maurício et al. [contribution 4] used HPLC coupled to a hybrid quadrupole Orbitrap MS for the identification of lipid species in edible *Chlorella vulgaris* strains, which allowed the identification of 316 lipid species, and Zong et al. [contribution 4] employed UHPLC coupled to a triple quadrupole time-of-flight mass spectrometer (Triple-TOF/MS) for the identification of 13 non-sugar compounds potentially linked to sugar degradation of inulin-type fructan and fructans after acid hydrolysis.

GC-MS has also undergone major developments in food analysis applications [1]. Maurício et al. [contribution 4] and Krusinski et al. [contribution 2] used this technique for the analysis of the fatty acid composition of *Chlorella vulgaris* strains and beef, respectively. Kim et al. [contribution 9] used the same methodology for the detection of 1,4-dioxane. Nevistić and Tomas [contribution 8] took advantage of GC-MS/MS to analyze more than 200 pesticide residues in different food matrices. As previously mentioned, GC can also be used for assessing the organoleptic quality of foods. In this regard, Carabetta et al. [contribution 1] and Wu et al. [contribution 5] analyzed aroma-active compounds in honey by GC-MS and in strawberry by GC-MS/MS, respectively.

Advances in mass spectrometry tools have been vast, and other techniques have emerged and are now fundamental in food analysis [8], as inductively coupled plasma-mass spectrometry (ICP-MS). Krusinski et al. [contribution 2] took advantage of this technique for the mineral analysis of beef. Indeed, ICP-MS is a leading technique in speciation analysis of elements, allowing low detection limits with good sensitivity and selectivity [8].

### 2.3. Trends in Analytical Extraction

Although some samples can be directly analyzed with minimal preparation (e.g., simple dilution and filtration), a more complex multistage extraction procedure is frequently needed before analysis [1]. The degradation of the compounds during extraction [contribution 4] and matrix effects [contributions 7,8] can lead to inaccuracies in the results.

All the articles published in this SI underlined the need for sample preparation and extraction. For instance, the modified Folch’s method [contribution 3] and microwave-assisted extraction [contribution 2] were used for the extraction of lipids, followed by a esterification procedure to obtain FA methyl esters (FAMEs) [contributions 2,3]; an acidic hydrolysis followed by solid-phase extraction (SPE) was used for the analysis of inulin-type fructans monosaccharides [contribution 4]; a multi-step preparation of honey samples [contribution 1] and Thai basil powder extracts [contribution 6] was conducted before the amino acid and polyphenolic analysis, respectively; a solvent extraction by ultrasonication, followed by alkaline and liquid–liquid extraction, was performed for the analysis of free, conjugated, and bound phenolic compounds [contribution 5]; and the extraction of fat-soluble vitamins and polyphenols in beef were performed using two different methods [contribution 2].

Nevistić & Tomas [contribution 8] investigated the matrix effects of more than 200 pesticide residues in four food matrices by GC coupled to tandem mass spectrometry (MS/MS) using QuEChERS (Quick, Easy, Cheap, Effective, Rugged, and Safe) sample preparation, which has been widely employed in food analysis, specifically in the quantitation of xenobiotics [1,2]. The results emphasized the relevance of using matrix-matched calibration curves for the monitoring of pesticide residues in feed and food. Similarly, Yévenes [contribution 7] employed a sample preparation methodology involving the extraction of antimicrobials in lettuce, followed by dispersive solid-phase extraction (dSPE) for sample clean-up using matrix-matched calibration curves.

When the main goal is the analysis of aroma-active compounds by GC-MS, headspace solid-phase microextraction (HS-SPME) is widely adopted as the standard extraction choice [1]. Carabetta et al. [contribution 1] used HS-SPME for the analysis of aroma-active compounds in honey. The static headspace (SH) method, which uses a stationary vapor phase [1], was used for the detection of 1,4-dioxane [contribution 9].

## 3. Future Perspectives

The nine articles published in this SI gather some of the latest research and advancements in food analysis, addressing the application of chromatography and mass spectrometry tools in several fields of food science. The improvements on these techniques have been enhancing the throughput of food quality and safety assessments, allowing the analysis of bioactive compounds and harmful contaminants in complex matrices, which are often present at trace levels [2,5]. Furthermore, emerging high-resolution tandem mass spectrometry techniques alongside the use of chemometrics have been recognized as a promising approach for innovation in food safety and authenticity assessment [5,6]. The identification of specific chemical markers and the development of universal and validated models are essential for reliable authentication [5].

The higher levels of sensitivity have been allowing simplified sample preparation protocols [5], however, sample preparation is still required in several cases, particularly for complex food matrices. Efforts continue to be directed towards on simplifying and improving sample handling protocols to ensure reproducible and accurate results [1,2,4].

Over the foreseeable future, it is expected that targeted and untargeted analyses will not be seen as mutually exclusive. In several cases, the combination of two or more analytical techniques, as low and high mass resolution, may make the best use of their advantages [6]. Current trends suggest that the synergy between the improvement of chromatographic columns and MS technological advances will boost the development of these techniques [2,3]. The research presented in this SI will serve as a basis for future studies in food analysis, significantly contributing to this evolving field.

## Figures and Tables

**Table 1:** Summary of the published articles in SI and main outcomes achieved. C: Contributions.

Sample	Goal	Chromatographic Technique	Main Outcomes	C
Calabrian unifloral honeys	To develop a predictive model correlating amino acids to volatile aroma compounds.	UHPLC-ESI-MS/MS (Triple Quad MS) HS-SPME-GC-MS	A predictive model correlating the amino acid profile to volatile aroma compounds of different varieties of honey was built. A strong linear association was found between specific amino acids and various classes of volatile compounds. The study demonstrated significant potential as a modern analytical tool for the rapid multicomponent analysis of food.	1
Beef samples resulting from cattle fed with: Complex pasture mixture (GRASS). Total mixed feedlot ration (GRAIN) Total mixed feedlot ration + 5% grape seed extract (GRAPE).	To compare fatty acid, micronutrient, and phytochemical composition.	GC-MS; UPLC-MS/MS (Triple Quad MS)	GRASS beef showed a lower ω-6:ω-3 ratio and higher levels of long-chain ω-3 polyunsaturated fatty acids (PUFAs), minerals, and secondary metabolites. For total ω-6 PUFAs, C20:3 *n*-3, and docosahexaenoic acid, the GRAPE samples exhibited intermediate values when compared to the GRASS and GRAIN groups. The finishing diet of cattle can be determined by analyzing the total PUFAs.	2
Autotrophic (C-Auto) and heterotrophic (C-Hetero) *Chlorella vulgaris* strains Chlorophyll-deficient mutants (C-White and C-Honey)	To assess fatty acid (FA) and lipid profile; to evaluate their antioxidant and anti-inflammatory properties.	GC-FID; LC-MS/MS (hybrid quadrupole Orbitrap MS)	C-Auto has the highest lipid content and ω-3 polyunsaturated fatty acids (PUFAs), and a higher content of glycolipids esterified to ω-3 PUFAs. C-Hetero, C-Honey, and C-White had higher levels of ω-6 PUFAs. C-White showed a higher content of phospholipids. C-Hetero and C-Honey had a higher content of triacylglycerols. All strains showed antioxidant and anti-inflammatory activities.	3
Inulin-type fructan (ITF)	To maximize the recovery of monosaccharides and find non-sugar byproducts from the hydrolysis of ITF by optimizing the acid hydrolysis conditions (acid concentration, temperature, and time).	UPLC-Triple-TOF/MS	Fructose was more susceptible to degradation compared to glucose, and milder hydrolysis conditions were more suitable for preserving the integrity of ITF. Furfural, 5-hydroxymethyl-2-furaldehyde, and 5-methyl-2-furaldehyde were associated with fructose degradation as the severity of hydrolysis conditions increased.	4
Geographical indication certified (GI) and non-GI strawberries	To compare the aroma and phenolic acid composition.	GC–MS UHPLC–MS/MS (Triple Quad MS)	GI strawberries are distinguished from non-GI strawberries by having higher concentrations of certain esters/ketones (e.g., isoamyl butyrate, trans-2-octen-1-ol) responsible for the specific aroma. The total phenolic content ranged from 24.41 to 36.46 mg/kg of fresh weight. Cinnamic acid could be used as a phenolic acid marker of GI strawberries. Chemometric techniques, such as OPLS–DA a small panel of indicators may accurately distinguish GI from non-GI strawberries.	5
Water, ethanol, and ethyl acetate extracts of Thai basil: *Ocimum basilicum* var. thyrsiflorum and *O*. *basilicum* cv.Jumbo 4320	To evaluate the phytochemical profile and antioxidant, antimicrobial, antithrombotic, and cytotoxic activities.	HPLC-PDA	Chlorophyll a, chlorophyll b, pheophytin a, and pheophytin b were quantitated in both ethanol and ethyl acetate extracts; ethanol extracts exhibited a higher concentration of these pigments. Gallic acid, catechin, apigenin, caffeic, coumaric, and sinapic acids were identified in both water and ethanol extracts. Ethyl acetate was only an effective solvent for extracting gallic acid. Both Thai basil varieties are potential sources of functional food ingredients with diverse bioactivities, depending on the extraction solvent.	6
Lettuce	To implement and validate a method for the detection of residues of oxytetracycline (OTC), 4-epi-oxitetracycline (4-epi-OTC), enrofloxacin (EFX), sulfachloropyridazine (SCP), and ciprofloxacin (CFX), and the detection of these drug residues in commercially available lettuce.	HPLC-MS/MS (QTRAP MS)	The method was validated by calculating parameters such as the specificity, linearity, recovery, precision, limit of detection (LOD), and limit of quantitation (LOQ). The method achieved LOD (0.8 µg·kg^−1^ dw for OTC, 4-epi-OTC, CFX, and EFX; 4.5 µg·kg^−1^ dw for EFX) and LOQ (1 µg·kg^−1^ dw for OTC, 4-epi-OTC, CFX, and EFX; 5 µg·kg^−1^ dw for EFX) that are suitable for regulatory surveillance in plant-based foods. Trace levels of OTC and EFX residues were detected in commercial lettuce from the main markets of Santiago (Chile).	7
Apples, grapes, spelt kernels, and sunflower seeds	To determine the matrix effects during the analysis of more than 200 pesticide residues using QuEChERS sample preparation.	GC-MS/MS	Both signal suppression and enhancement were observed for all matrices, and their degree was dependent on the analyte/matrix combination. For high water content samples (apples and grapes) strong signal enhancement was observed for the majority of pesticides. For high starch and/or protein content, high oil and low water content (spelt kernels and sunflower seeds), signal suppression was the most common.	8
Polyethylene glycol 600 (PEG 600)	To develop and validate a method for quantitating trace 1,4-dioxane in food additive matrices.	Static headspace (SH) GC-MS	The optimal sample pretreatment and analytical conditions for SH-GC-MS were determined. The method was validated and met the requirements and standards of the Ministry of Food and Drug Safety’s standard procedure guidelines for the food additive inspection.	9
